# Correction to “Multicomponent Approach for Stable Methylammonium-Free Tin–Lead Perovskite Solar Cells”

**DOI:** 10.1021/acsenergylett.4c02514

**Published:** 2024-10-02

**Authors:** Silver-Hamill Turren-Cruz, Jorge Pascual, Shuaifeng Hu, Jesus Sanchez-Diaz, Sergio Galve-Lahoz, Wentao Liu, Wolfram Hempel, Vladimir S. Chirvony, Juan P. Martinez-Pastor, Pablo P. Boix, Atsushi Wakamiya, Iván Mora-Seró

The authors regret an error in the Acknowledgments section of the original publication: the first sentence of the Acknowledgments was incomplete. The corrected first sentence should read as follows:

“S.-H.T.-C would like to thank the Ministry of Science and Innovation of Spain (postdoctoral contract Juan de la Cierva Formación FJC2019-041835-I), Generalitat Valenciana under BEST project (CIBEST/2021/234) and POLONEZ BIS project No. 2021/43/P/ST5/01780, co-funded by the National Science Centre and the EU’s H2020 research and innovation programme under the MSCA 945339, for the financial support during this work.”

This revision does not affect the main text and the conclusion of the published article. The authors would like to apologize for any inconvenience caused.

